# Prevalence, Determinants, and Consequences of Vestibular Hypofunction. Results From the KORA-FF4 Survey

**DOI:** 10.3389/fneur.2018.01076

**Published:** 2018-12-07

**Authors:** Eva Grill, Maria Heuberger, Ralf Strobl, Murat Saglam, Rolf Holle, Birgit Linkohr, Karl-Heinz Ladwig, Annette Peters, Erich Schneider, Klaus Jahn, Nadine Lehnen

**Affiliations:** ^1^Institute for Medical Information Processing, Biometrics and Epidemiology, Ludwig-Maximilians Universität München, Munich, Germany; ^2^German Center for Vertigo and Balance Disorders, Ludwig-Maximilians Universität München, Munich, Germany; ^3^Munich Center of Health Sciences, Ludwig-Maximilians Universität München, Munich, Germany; ^4^Department of Neurology, University Hospital Munich, Ludwig-Maximilians Universität München, Munich, Germany; ^5^German Research Center for Environmental Health (GmbH), Institute of Health Economics and Health Care Management, Helmholtz Zentrum München, Neuherberg, Germany; ^6^German Research Center for Environmental Health (GmbH), Institute of Epidemiology, Helmholtz Zentrum München, Neuherberg, Germany; ^7^Department of Psychosomatic Medicine and Psychotherapy, Klinikum Rechts der Isar, Technical University of Munich, Munich, Germany; ^8^Institute for Medical Informatics, Brandenburg University of Technology Cottbus—Senftenberg, Senftenberg, Germany

**Keywords:** prevalence, vertigo, vestibular hypofunction, aged, head impulse test, Video-HIT

## Abstract

**Objective:** Uni- or bilateral vestibular hypofunction (VH) impairs balance and mobility, and may specifically lead to injury from falls and to disability. The extent of this problem in the general population is still unknown and most likely to be underestimated. Objective of this study was to determine the prevalence, determinants, and consequences of VH in the general population.

**Methods:** Data originates from the cross-sectional second follow-up (FF4) in 2013/14 of the KORA (Cooperative Health Research in the Region of Augsburg)-S4 study (1999–2001) from Southern Germany. This was a random sample of the target population consisting of all residents of the region aged 25–74 years in 1999. We included all participants who reported moderate or severe vertigo or dizziness during the last 12 months and a random sub-sample of participants representative for the general population without vertigo or dizziness during the last 12 months were tested. VH was assessed with the Video-Head Impulse Test (vHIT). Trained examiners applied high-acceleration, small-amplitude passive head rotations (“head impulses”) to the left and right in the plane of the horizontal semicircular canals while participants fixated a target straight ahead. During head impulses, head movements were measured with inertial sensors, eye movements with video-oculography (EyeSeeCam vHIT).

**Results:** A total of 2,279 participants were included (mean age 60.8 years, 51.6% female), 570 (25.0%) with moderate or severe vertigo or dizziness during the last 12 months. Of these, 450 were assessed with vHIT where 26 (5.8%) had unilateral VH, and 16 (3.6%) had bilateral VH. Likewise, 190 asymptomatic participants were tested. Of these 5 (2.6%) had unilateral VH, and 2 (1.1%) had bilateral VH. Prevalence of uni- or bilateral VH among tested symptomatic participants was 2.4% in those < 48 years, and 32.1% in individuals aged 79 and over. Age-adjusted prevalence was 6.7% (95% CI 4.8%; 8.6%). VH was associated with worse health, falls, hearing loss, hearing impairment, and ear pressure.

**Conclusion:** VH may affect between 53 and 95 million adults in Europe and the US. While not all affected persons will experience the full spectrum of symptoms and consequences, adequate diagnostic and therapeutic measures should become standard of care to decrease the burden of disease.

## Introduction

Vestibular hypofunction (VH) is a partial or complete deficit of function of the peripheral or central vestibular system. While VH may have traumatic, toxic, infectious, genetic, and neurodegenerative causes, etiology is in about 50% of cases unknown (1). As vestibular input is needed for gaze stabilization, dynamic stability of gait, and for spatial orientation during locomotion, uni- or bilateral VH has many direct and indirect consequences on functioning and daily life.

The most frequent consequences include chronic dizziness with or without vertigo, oscillopsia, and problems with balance, walking and driving (1, 2). Patients may e.g., not be able to read signs while moving, may fall more often, or have difficulties walking in the dark or on uneven surfaces. Moreover, there is conclusive evidence that VH impairs spatial memory, learning, and wayfinding (3); its effects on higher cognitive functions (4), on social cognition (5) and on cardiovascular regulation (6) are topics of ongoing research. This is why patients with VH report considerable negative impact on social participation and quality of life (7, 8). Generally, persons with bilateral VH are more severely affected than those with unilateral VH.

When regarding the consequences on quality of life and the considerable burden of disease, it seems surprising that VH as an impairment of a basic sensory system does not get the same attention as e.g., the impaired auditory or visual system. One major issue is that the prevalence of VH and therefore its impact on a population level is assumed to be relatively low. For bilateral VH literature reports a frequency of ~500,000 persons in Europe and in the US (7, 8). However, as patients present with a large variety of symptoms, reliably analyzing the prevalence of VH in the general population based on symptom constellation without performing gold-standard clinical tests is a challenge and most likely underestimates the true frequency and relevance. More reliable estimates are therefore needed to gain insights into this pathology and its true consequences.

The objective of this study was to determine the prevalence and determinants of uni- and bilateral vestibular hypofunction (VH )in the general population based on a representative sample and to examine its consequences on self-reported health of the affected individuals.

## Materials and Methods

### Study Design and Participants

Data originates from the KORA FF4 study (Cooperative Health Research in the Region of Augsburg). The KORA FF4 study is the second follow-up of the KORA S4 study, a population-based health survey conducted in the city of Augsburg and two surrounding counties between 1999 and 2001. A total sample of 6,640 persons was drawn from the target population consisting of all residents of the region aged 25–74 years.

Of all 4,261 participants of the S4 baseline study 2,279 also participated in the 14-years follow-up FF4 study. The follow-up study was conducted from 03/06/2013 to 27/09/2014. Persons were considered ineligible for FF4 if they had died in the meantime (*n* = 455, 10.7%), lived too far outside the study region or were completely lost to follow-up (*n* = 296, 6.9%), or had demanded deletion of their address data (*n* = 191, 4.5%). Of the remaining 3,319 eligible persons, 157 could not be contacted, 504 were unable to participate because they were too ill or had no time, and 379 were not willing to participate in this follow-up, giving a response rate of 68.7%.

Variables were either collected through telephone or face-to-face interview or through direct measurement at the study center. The investigations were carried out in accordance with the Declaration of Helsinki, including written informed consent of all participants. All study methods were approved by the ethics committee of the Bavarian Chamber of Physicians, Munich (FF4: EC No. 06068).

### Measures: Vestibular Hypofunction

Presence of vertigo and dizziness, as well as falls, was assessed using standardized questions from the balance section of the National Health and Nutrition Examination Survey (NHANES) questionnaire (see http://www.cdc.gov/nchs/nhanes/nhanes2003-2004/BAQ_C.htm) in the face to face interview. If the initial question on lifetime vertigo was affirmed it was followed by a question on vertigo during the last 12 months and on falls. To assess the presence or absence of VH, all participants who reported moderate or severe vertigo and dizziness during the last 12 months were tested with the Video-Head Impulse Test (vHIT). To estimate the prevalence of VH in asymptomatic persons, we also tested a random sub-sample of participants who were representative for the general population and who reported no vertigo during the last 12 months.

Head impulse testing (HIT) is the neuro-otological standard for assessing vestibulo-ocular reflex function (9). The HIT is conducted by introducing high-acceleration, small-amplitude passive head rotations (“head impulses”) to the left and right in the plane of the horizontal semicircular canals while participants fixate a target straight ahead. Eye movements are monitored during this passive head movement. Vestibular deficit causes re-fixation problems of the eye which can be observed as eye saccades. The HIT is mostly straightforward when conducted by an experienced clinician; however, reliable detection of saccades can be a challenge. For objective detection of saccades and their quantification we therefore used Video-head impulse testing (vHIT) with the EyeSeeCam System (10, 11). During head impulses, head movements are measured with inertial sensors, and eye movements are recorded with video-oculography. These recordings allow a standardized assessment of the re-fixation saccades after the impulse. To determine the presence or absence of VH, the gain of the vestibulo-ocular reflex was calculated as the ratio of the median of eye and head velocity in a window between 55 and 65 milliseconds (ms) after head impulse start. Three examiners without any prior experience in head impulse testing were trained in vHIT testing. During the observation time of 16 months that also included training and a pilot period, data quality was checked weekly by experienced neuro-otologists; examiners were repeatedly retrained individually if necessary. Technical errors leading to insufficient vHIT quality were noted and reported back to the examiners. Persons with known acute problems of the cervical spine, e.g., wearing a cervical collar or having experienced a cervical disc herniation, and people experiencing pain with small twists of the head were excluded from the vHIT. Usability and validity of the procedures have been described previously (12).

The outcome of the vHIT measurement was rated by two experienced oto-neurologists and categorized as either “none,” “unilateral,” or “bilateral” hypofunction. Raters were blinded to symptom status. The category hypofunction was assigned if the gain of the vestibular-ocular reflex was <0.79 and re-fixation saccades were present (13, 14).

### Measures: Outcomes

A single-item question was used to measure self-rated health (options: very good, good, rather bad, bad). Self-rated health in comparison with others was measured using the question “How would you rate your current health status in comparison to others of the same age?” (options: better, equal, worse).

### Measures: Covariates

For the purpose of the analyses, age was defined as age at reference date (July 1st 2014) and age groups were defined accordingly. Information on education, marital status, alcohol consumption, physical activity, self-rated health, and morbidity, were collected by self-report in the interview. The information on education was obtained in the baseline S4 survey where participants provided their highest level of school qualification.

According to the German school system, the standard educational level includes participants with up to 9 years of schooling. Medium educational level is equivalent to 10 years of schooling and high educational level to 12 or 13 years of schooling, required to enter a university (15).

BMI was calculated as body weight in kilograms measured at the study center divided by squared height in meters. BMI was categorized as underweight (BMI < 18.5), normal weight (18.5 ≤ BMI < 25.0), overweight (25.0 ≤ BMI < 30), obesity grade I (30 ≤ BMI < 35), obesity grade II (35 ≤ BMI < 40), and obesity grade III (BMI ≥ 40).

Leisure time physical activity was assessed with two separate questions concerning leisure time sport activity in winter and in summer (cycling included) and was categorized into inactive (“No activity” and “Low activity”) and active (“Moderate activity” and “High activity”) (16).

Data on health conditions included self-reported of hearing loss, hearing impairment, ear pressure and ear noises, cancer diagnosed in the past 3 years, diabetes, angina pectoris, myocardial infarction, and stroke (17).

Blood pressure and resting heart rate was measured after a rest period of at least 5 min in a sitting position and repeated three times at an interval of 3 min with a standardized protocol (18). Hypertension was classified according to the 1999 World Health Organization-International Society of Hypertension Guidelines for the management of hypertension (19) into six classes: optimal (< 120/80 mmHG), normal (120/80–130/85 mmHG), high normal(130/85–140/90 mmHG), hypertension grade I (140/90–160/100 mmHG), hypertension grade II (160/100–180/110 mmHG), and hypertension grade III (≥180/110 mmHG).

### Statistical Analysis

Categorical variables were described with percentages and numeric variables with means. We tested for bivariate differences between groups of participants using Chi-squared tests for categorical and *t*-tests for continuous variables.

To investigate the impact of VH on self-reported health we used logistic regression. For this purpose we assumed that all asymptomatic persons who were not tested with vHIT were without pathological vestibular findings.

Model fit was tested by the Hosmer-Lemeshow statistic, which should be non-significant (*p* > 0.05) to maintain the null hypothesis of adequate fit (20). We tested for collinearity using the Variance Inflation Factor (VIF) and for logit-linearity using Box-Tidwell tests.

Prevalence of VH in the general population was estimated using the available information of persons with vHIT stratified for persons aged under 70 or aged 70 and over. Confidence intervals for the number of persons and for percentages were calculated using the standard formula:

$$\begin{array}{l} KI\left(P\right)=P\pm z_{1-\alpha /2}\sqrt{Var\left(P\right)} \end{array}$$

The joint variance prevalence estimates for younger and older participants was calculated according to:

$$\begin{array}{l} Var(P)=Var(\frac{\left(P_{1}\cdot n_{1}+P_{2}\cdot n_{2}\right)}{N})\\ \;=Var(P_{1}\cdot \frac{n_{1}}{N}+P_{2}\cdot \frac{n_{2}}{N})=\\ \;={\left(\frac{n_{1}}{N}\right)}^{2}\cdot Var(P_{1})+\;{\left(\frac{n_{2}}{N}\right)}^{2}\cdot Var(P_{2})=\\ \;={\left(\frac{n_{1}}{N}\right)}^{2}\cdot \frac{P_{1}\cdot (1-P_{1})}{n_{1}^{*}\;}+\;{\left(\frac{n_{2}}{N}\right)}^{2}\cdot \frac{P_{2}\cdot (1-P_{2})}{n_{2}^{*}} \end{array}$$

with *P*_1_ and *P*_2_ as the prevalence estimates of each group, *n*_1_ and *n*_2_ the total number in each group, $n_{1}^{*}$ and $n_{2}^{*}$ the number of examined persons in each group, and *N* as the sum of *n*_1_ and *n*_2_.

R 3.0.3 was used for all analyses (21). Statistical significance was set at a two-tailed 5% level.

## Results

**Figure 1:**
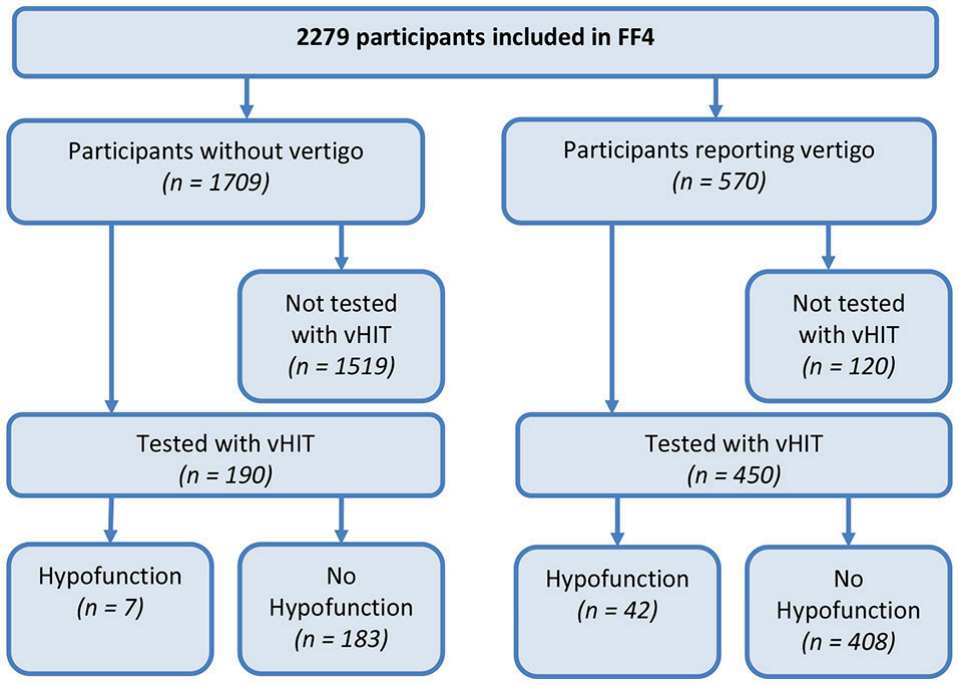
Flow diagram of participants in the KORA FF4-study assessed with Video-Head Impulse Testing (vHIT). Vertigo refers to moderate or severe vertigo or dizziness during the last 12 months. Vestibular hypofunction on vHIT was defined as a gain of the vestibular-ocular reflex <0.79 and detectable re-fixation saccades.

A total of 2,279 participants were included (mean age 60.8 years, range 39–88, 51.6% female), 813 reported life-time vertigo or dizziness (35.7%) and 570 (25.0%) reported moderate or severe vertigo or dizziness during the last 12 months. Of these, 18.2% participants were not eligible for the vHIT. Due to technical problems 2.8% vHIT recordings could not be analyzed. Thus, 450 participants who reported vertigo or dizziness in the last 12 months could be assessed with the vHIT. Of these 450 symptomatic participants 5.8% had unilateral VH, and 3.6% had bilateral VH. Also, 233 asymptomatic participants representative for the study population were randomly chosen for testing. Of these, 16.3% participants were not eligible for the vHIT, and 2.1% vHIT recordings could not be analyzed due to technical problems. Of the tested 190 asymptomatic participants 2.6% had unilateral VH, and 1.1% had bilateral VH. Participant flow is shown in Figure 1.

Prevalence of uni- or bilateral VH among tested symptomatic participants increased with age, from 2.4% in individuals younger than 48 years to 32.1% in individuals aged 79 and over. Prevalence of bilateral VH in symptomatic participants aged 79 and over was 16.1%. Table 1 shows the characteristics of participants stratified for presence of VH. Persons with VH were more likely to report severely impaired health, more falls, hearing loss, hearing impairment or ear pressure, and were significantly older. Likewise, persons with VH rated their own health worse in comparison to others of the same age.

**Table 1:** Characteristics of participants stratified by presence or absence of vertigo and vestibular hypofunction (VH).

	**Without vertigo^a^**	**With vertigo^a^**		**Without vs. VH**	**VH vs. Non-VH**
**(No. with data)**	**(*****n*** **=** **1709)**	**VH (*****n*** **=** **42)**	**Non-VH (*****n*** **=** **408)**	***p*****-value^b^**	***p*****-value^b^**
Female	823 (48.2%)	26 (61.9%)	242 (59.3%)	0.0279	0.3896
**AGE**					
39–48	382 (22.4%)	2 (4.8%)	81 (19.9%)	< 0.0001	< 0.0001
49–58	437 (25.6%)	3 (7.1%)	99 (24.3%)		
59–68	416 (24.3%)	8 (19.0%)	105 (25.7%)		
69–78	340 (19.9%)	11 (26.2%)	85 (20.8%)		
79–88	134 (7.8%)	18 (42.9%)	38 (9.3%)		
**EDUCATIONAL LEVEL**					
Standard	807 (47.3%)	27 (64.3%)	209 (51.5%)	0.5267	0.2468
Medium	437 (25.6%)	6 (14.3%)	106 (26.1%)		
High	463 (27.1%)	9 (21.4%)	91 (22.4%)		
**BMI**					
Underweight	8 (0.5%)	0 (0%)	2 (0.5%)	0.1475	0.4647
Normal weight	517 (30.3%)	10 (23.8%)	124 (30.5%)		
Overweight	722 (42.3%)	17 (40.5%)	164 (40.3%)		
Obesity grade I	329 (19.3%)	10 (23.8%)	81 (19.9%)		
Obesity grade II	90 (5.3%)	1 (2.4%)	20 (4.9%)		
Obesity grade III	42 (2.5%)	4 (9.5%)	16 (3.9%)		
Falls in the last 12 months	489 (28.6%)	23 (54.8%)	171 (41.9%)	0.0007	0.1357
Physically active	1001 (58.6%)	19 (45.2%)	220 (53.9%)	0.479	0.9960
**ALCOHOL IN g/day**					
0	434 (25.4%)	16 (38.1%)	122 (29.9%)	0.3684	0.3926
[0; 20]	741 (43.4%)	15 (35.7%)	182 (44.6%)		
[20; 40]	314 (18.4%)	6 (14.3%)	65 (15.9%)		
[40; 60]	154 (9.0%)	5 (11.9%)	26 (6.4%)		
[60; 80]	37 (2.2%)	0 (0%)	5 (1.2%)		
> = 80	28 (1.6%)	0 (0%)	8 (2.0%)		
**SMOKING**					
Never	768 (44.9%)	24 (57.1%)	185 (45.3%)	0.6737	0.3288
Former	665 (38.9%)	13 (31.0%)	164 (40.2%)		
Current	276 (16.1%)	5 (11.9%)	59 (14.5%)		
Hearing loss	148 (8.7%)	13 (31.0%)	53 (13.1%)	< 0.0001	0.0039
Hearing impairment	281 (16.5%)	19 (45.2%)	108 (26.7%)	0.0029	0.2904
Ear pressure	159 (9.3%)	14 (33.3%)	80 (19.6%)	< 0.0001	0.0136
Ear noises	489 (28.6%)	23 (54.8%)	171 (41.9%)	0.0007	0.1357
**BLOOD PRESSURE**					
optimal	896 (52.5%)	21 (50.0%)	219 (53.7%)	0.597	0.8855
normal	356 (20.9%)	9 (21.4%)	77 (18.9%)		
high normal	221 (12.9%)	8 (19%)	63 (15.4%)		
Hypertension grade 1	178 (10.4%)	3 (7.1%)	43 (10.5%)		
Hypertension grade 2	44 (2.6%)	1 (2.4%)	4 (1.0%)		
Hypertension grade 3	12 (0.7%)	0 (0%)	2 (0.5%)		
Resting heart rate > = 70	900 (52.7%)	17 (40.5%)	221 (54.2%)	0.2006	0.2181
Myocardial Infarction	59 (3.5%)	1 (2.4%)	15 (3.7%)	0.1865	0.3009
Angina Pectoris	78 (4.6%)	1 (2.4%)	36 (8.8%)	0.2975	0.1324
Diabetes	146 (8.5%)	9 (21.4%)	43 (10.5%)	0.2060	0.5203
Stroke	36 (2.1%)	4 (9.8%)	9 (2.2%)	0.1644	0.2282
Cancer	186 (10.9%)	7 (16.7%)	47 (11.5%)	0.8998	0.7098
Self-rated health rather bad/bad	266 (15.6%)	22 (52.4%)	115 (28.2%)	< 0.0001	0.1075

a self reported moderate or severe vertigo or dizziness in the last 12 months.

b *adjusted for age and sex*.

The association of VH with self-rated health was still significant after adjusting for covariates (see Table 2).

**Table 2:** Multivariable adjusted association of vestibular hypofunction and measures of self-rated health (*n* = 2279).

		**Self-rated health^a^**			**Health in comparison to others^b^**		
		**OR**	**CI**	***p*****-value**	**OR**	**CI**	***p*****-value**
Vestibular hypofunction		2.11	[1.12; 3.90]	0.0185	1.92	[1.03; 3.65]	0.0424
Female		1.57	[1.26; 1.96]	< 0.0001	1.68	[1.41; 2.00]	< 0.0001
**Age**	**Under 49**	**Reference**			**Reference**		
	49–58	1.56	[1.08; 2.28]	0.0201	0,58	[0.44; 0.75]	< 0.0001
	59–68	2.21	[1.54; 3.19]	< 0.0001	0.40	[0.30; 0.52]	< 0.0001
	69–78	2.32	[1.60; 3.40]	< 0.0001	0.30	[0.23; 0.40]	< 0.0001
	79–88	3.72	[2.43; 5.75]	< 0.0001	0.19	[0.13; 0.28]	< 0.0001
**Education**	**Low**	**Reference**			**Reference**		
	Medium	0.76	[0.58; 0.99]	0.0466	0.78	[0.63; 0.97]	0.0246
	High	0.62	[0.46; 0.83]	0.0013	0.80	[0.64; 0.99]	0.0407
Ear pressure		2.30	[1.73; 3.05]	< 0.0001	1.50	[1.15; 1.97]	0.0032

Models were additionally adjusted for Body Mass Index, hypertension, cancer diagnosed in the past 3 years, diabetes, angina pectoris, myocardial infarction, and stroke. Odds Ratios >1 indicate worse health.

a “How would you rate your current health status?” Options very good/good, vs. rather bad/bad.

b “How would you rate your current health status in comparison to others of the same age?” Options better vs. equal/worse.

*OR, Odds Ratio; CI, 95% Confidence Interval*.

Using these results and assuming that eligibility for Video-HIT was independent of vestibular status, age-adjusted prevalence of bilateral VH in the general adult population aged 39 years or older is 2.5% (95% confidence interval [1.4%; 3.7%]). Under the same assumptions, the age-adjusted prevalence of uni- or bilateral VH in the general population is 6.7% [4.8%; 8.6%].

## Discussion

Based on objective clinical testing we could show that VH is rather common in the general population and may occur, albeit much less frequently, also in individuals who do not report distinct symptoms of vertigo or dizziness. In our study, persons with VH reported worse health, even when adjusted for health status, and more falls.

With a prevalence of over 5% of the adult population the results from our study exceed previous much lower estimates. A study in the adult US population that based the diagnosis of VH on a constellation of self-reported symptoms and patient history found a prevalence of 28/100,000 (8). A nationally representative survey estimated a 1 year prevalence of vestibular disease of 4.9% based on symptoms (22), but also without technically ascertaining vestibular function.

It is notable that according to our findings over 25% of symptomatic older adults can be expected to have VH. This is in agreement with studies investigating the age-related degeneration of the vestibular system (23) which find that vestibular-ocular reflex gain decreases with increasing age (24). These degenerative processes are likely to be due to genetic predisposition and cumulative environmental factors over the life span such as exposure to noise, infections, toxic agents, and air pollutants (25). It seems plausible that risk factors and damage to the inner ear, e.g., as a consequence of Menière's disease, and neurodegenerative processes, accumulate with age (26). This idea is further confirmed by our finding that hearing loss and hearing impairment were significantly more frequent in individuals with VH, indicating e.g., previous instances of Menière's disease.

However, the findings of the current study do not support previous studies that found an association of diabetes with vestibular disease (8, 27). In a representative sample of US adults aged 40 years and older 54% of individuals with diabetes mellitus had any kind of vestibular dysfunction (27) as compared to 27% in our study with any vertigo (52 of 198 individuals with diabetes) and 5% (9 of 198) with uni- or bilateral VH. This difference might again be attributable to the different methods of establishing the diagnosis of VH. Also, the number of persons with VH found in our sample might not be large enough to make any relevant conclusions about the association to diabetes mellitus. As pathophysiological mechanisms of this association seem plausible, further studies are recommended.

Our data showed that persons with VH fall more often. A high prevalence of VH in older symptomatic adults does have implications for the prevention of imbalance and calls for a more profound approach toward the diagnosis of balance problems in this group.

In line with others (22) we could show that vertiginous symptoms were more frequent in women. Recent research has suggested that e.g., vestibular migraine, a disease entity with higher prevalence in women, is associated with hormonal receptor status (28). Also, Menière's disease seems to be more frequent in women (29). However, in contrast to earlier findings (8, 30) our study could not confirm a clear female preponderance of VH. If VH is predominantly attributable to degenerative loss of vestibular function, invariance to sex might be plausible. Persons with VH in our study certainly represent a mixture of all potential etiologies, but with a mean age of 60 years participants are more likely to have experienced deficit accumulation of the vestibular system. Still, the role of sex in vestibular disease and specifically in VH remains to be investigated more in detail.

Not surprisingly, persons with VH in our study reported worse health status than persons without VH. The negative impact of vertigo and dizziness on activities of daily living and quality of life has been shown consistently (31–33). It is interesting to note that self-reported health was worst among those individuals who had both symptoms of vertigo and objectively confirmed VH. There are reports stating that some patients adjust well to oscillopsia (34), however, the need for therapy is mostly unchallenged. Bilateral VH is a chronic condition where patients are not likely to improve (2), and where considerable economic burden and consequences for quality of life are to be expected (35). Vestibular rehabilitation has confirmed effectiveness in peripheral VH (36); while vestibular rehabilitation still needs to gain better attention and implementation in some countries (37) more experimental approaches are under development, e.g., external stimulation (38, 39) and efforts to design vestibular implants (7).

One of the major strengths of our study is the rigorous approach to objective vestibular testing. Our study showed that vestibular testing conducted by examiners without previous experience is challenging but feasible if a high level of quality control is maintained. Video-Head-Impulse-Testing provided a standardized way to examine VH in a large-sample survey setting. Yet, it has to be mentioned as a limitation of this method that part of the participants could not be tested due to existing problems of the cervical spine and neck. There is potential for selection bias. However, distribution of symptom severity and characteristics of this subgroup revealed that there were no substantial differences to the main group (data not shown), indicating a strong possibility that our prevalence estimates are valid or even underestimate the true frequency of VH. Another point to consider is that a very small proportion of persons who did not report any vertigo also tested positive for VH. It still needs to be determined if these were false positive results or if there is potential for full recovery from symptoms. Furthermore, the cross sectional study design does not allow any causal conclusions. Confirmed cases have to be followed-up to get a clear picture about long-term consequences and prognosis.

Vestibular hypofunction is an underestimated chronic pathology. Assuming an age-adjusted prevalence of 5 to 9%, i.e., the lower and upper confidence limits of our estimate, this condition may affect between 53 and 95 million adults in Europe and the US. While not all affected persons will experience a complete loss of vestibular function and the full spectrum of symptoms and consequences, adequate diagnostic, and therapeutic measures should become standard of care to decrease the burden of disease.

## Author Contributions

EG, MH, MS, ES, KJ, and NL contributed to the conception and design of the study. BL, RH, and AP organized data collection and the database. RS, MH, RH, and NL performed the statistical analysis. EG wrote the first draft of the manuscript. MH, NL, BL, RH, and AP wrote sections of the manuscript. All authors contributed to the manuscript revision, read and approved the submitted version.

### Conflict of Interest Statement

ES is a shareholder and general manager of EyeSeeTec GmbH, the manufacturer of EyeSeeCam vHIT, and unpaid consultant to Interacoustics AS, the distributor of EyeSeeCam vHIT. ES also received speaker honoraria from Actelion. NL is a shareholder and consultant to EyeSeeTec GmbH, the manufacturer of EyeSeeCam vHIT and received speaker honoraria from Interacoustics AS. The remaining authors declare that the research was conducted in the absence of any commercial or financial relationships that could be construed as a potential conflict of interest.
